# Modelling the Collective Mechanical Regulation of the Structure and Morphology of Epithelial Cell Layers

**DOI:** 10.3389/fcell.2022.767688

**Published:** 2022-03-24

**Authors:** Hamid Khataee, Madeleine Fraser, Zoltan Neufeld

**Affiliations:** School of Mathematics and Physics, The University of Queensland, Brisbane, QLD, Australia

**Keywords:** computational modeling, tissue modelling, mechanobiolgy, cell morphological analysis, cellular mechanics, stochastic modelling and simulation

## Abstract

The morphology and function of epithelial sheets play an important role in healthy tissue development and cancer progression. The maintenance of structure of closely packed epithelial layers requires the coordination of various mechanical forces due to intracellular activities and interactions with other cells and tissues. However, a general model for the combination of mechanical properties which determine the cell shape and the overall structure of epithelial layers remains elusive. Here, we propose a computational model, based on the Cellular Potts Model, to analyse the interplay between mechanical properties of cells and dynamical transitions in epithelial cell shapes and structures. We map out phase diagrams as functions of cellular properties and the orientation of cell division. Results show that monolayers of squamous, cuboidal, and columnar cells are formed when the axis of cell proliferation is perpendicular to the substrate or along the major axis of the cells. Monolayer-to-multilayer transition is promoted via cell extrusion, depending on the mechanical properties of cells and the orientation of cell division. The results and model predictions are discussed in the context of experimental observations.

## 1 Introduction

Understanding the mechanisms of the development of various tissue morphologies is a major challenge in biology (Hannezo et al., 2014). Epithelial cell layers are the simplest living tissues that line organs throughout the body (Vincent et al., 2015) and play important roles in regulating embryo development, yet account for about 90% of all cancers (Pedersen et al., 2013). Morphogenesis of organ systems is driven by the ability of cells to survive and proliferate (Chen et al., 1997; Streichan et al., 2014), primarily regulated by cell growth factors and cell-substrate adhesion (Chen et al., 1997; Schwartz and Assoian, 2001; Brakebusch et al., 2002).

For many adherent cells, cell proliferation can only occur on a substrate (Adam Hacking et al., 2013). The substrate maintains a dynamic force balance between the cell and its microenvironment, and thus, the loss of substrate or its abnormal stiffness can results in aberrant cellular behaviours, e.g., breast tumor progression (Provenzano and Keely, 2011). As feedback loops, cells sense the stiffness of their environment by pulling against the extracellular matrix, through integrin-extracellular matrix linkages, and/or neighbouring cells (Chen et al., 1997; Provenzano and Keely, 2011). This process is dependent on cell–substrate and cell-cell adhesion, as well as the contractility of cell cortex (Provenzano and Keely, 2011). Therefore, both integrins and growth factor receptors use cytoplasmic signaling pathways to regulate cell cycle progression and growth (Schwartz and Assoian, 2001). It has been shown that the probability of cell proliferation increases with increasing substrate stiffness (Mohan et al., 2018) and cell area (Streichan et al., 2014). Yet, it remains inconclusive how different forces and regulatory mechanisms within cells can affect proliferation orientation; reviewed in (Collinet and Lecuit, 2013; Finegan and Bergstralh, 2019).

Earlier theoretical studies on epithelial morphology have explored two-dimensional (2D) mechanical model of a tubular epithelium (Hočevar Brezavšček et al., 2012; Krajnc et al., 2013), geometric patterning of apical junctions (Gibson et al., 2006; Farhadifar et al., 2007; Käfer et al., 2007; Hilgenfeldt et al., 2008), shapes of cells and the buckling of cell monolayers (Osterfield et al., 2013; Hannezo et al., 2014). Although these models are based on the mechanical properties of cells, they were mostly restricted to monolayers. To model the dynamic processes involved in the formation of epithelial cell layers, models of epidermal homeostasis were proposed based on probabilistic rules associated to different types of cells (Doupé et al., 2010; Doupé et al., 2012; Kostiou et al., 2020). However, these models do not consider the shape of the cells and the role of cellular mechanics in modelling the transition between monolayers to multilayers. Therefore, it remains elusive how the mechanical properties of cells and their interactions determine cell aspect ratios and the formation of mono- and multilayered epithelial structures. Further, the role of the orientation of the plane of cell division, in combination with mechanical properties of cells, in modelling collective tissue morphology has not been explored.

Here, we propose a computational model for analysing the development of collective epithelial morphologies using the Cellular Potts Model (CPM) (Graner and Glazier, 1992; Glazier and Graner, 1993). CPM is a computational modelling framework that can represent the essential features of the real-world epithelial cell dynamics, and allows general predictions of the behaviour and morphology of cells (Khataee et al., 2020; Kempf et al., 2021). Our model simulates the transition of cell shapes and the formation of mono- and multilayered structures by altering various mechanical properties of identical proliferative cells.

### 2 Theoretical Model

To simulate the collective morphology of cells emerging through their mechanical properties and interactions on a substrate, we use a two-dimensional CPM (Graner and Glazier, 1992; Glazier and Graner, 1993) which represents a cross-section of cells on a substrate on a plane perpendicular to the substrate. The CPM is an on-lattice model which is computationally simpler than most off-lattice models, e.g., vertex model (Osborne et al., 2017; Giniūnait et al., 2019) and has been used to capture essential realistic features of epithelial cell dynamics (Kempf et al., 2021), e.g., the dynamics of cell migration on short microlanes (Zhou et al., 2020), circular micropatterns (Segerer et al., 2015), and in a confluent sheet expanding into a free region (Khataee et al., 2020).

**FIGURE 1:**
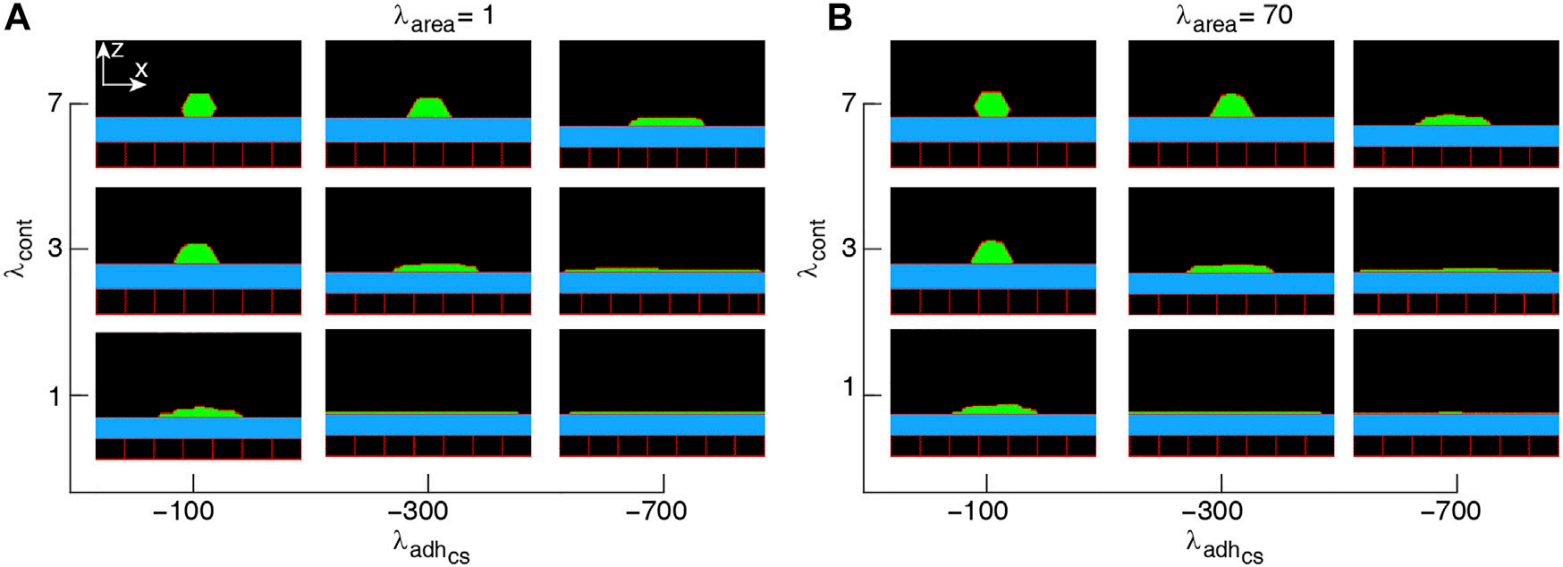
Phase diagram of single-cell shapes **(A,B)**. *x*-*z* cross-section of the cell (green) placed on a substrate (blue) surrounded by an empty region (black) and wall cells (black squares). Simulations were run with the cell proliferation disabled. The range of parameter values are adopted from (Khataee et al., 2020). Snapshots were taken in the steady-states.

The cells are represented on a lattice, where each cell covers a set of connected lattice sites (or pixels) and each pixel can only be occupied by one cell at a time. Here, the lattice is a rectangular surface (480 × 195 pixels in length and height, respectively), representing a cross-sectional view to epithelial cells placed on a substrate. This means that the model is 2D in the *x*-*z* plane, where *x* and *z* axes are parallel and perpendicular to the substrate, respectively (see inset in Figure 1A, top left corner). The expansion and retraction of the cell boundaries are determined by minimising a phenomenological energy *E*, defined in terms of the area *A*_*σ*_ and perimeter *L*_*σ*_ of each cell *σ* of *N* cells (indices *σ* = 1, … , *N*) (Farhadifar et al., 2007; Khataee et al., 2020; R. Noppe et al., 2015; Albert and Schwarz, 2016; Thüroff et al., 2019) as:

$$E={\text{λ}}_{\text{area}}\overset{N}{\underset{\sigma }{\sum }}{\left(A_{\sigma }-A_{\text{0}}\right)}^{2}+{\text{λ}}_{\text{cont}}\overset{N}{\underset{\sigma }{\sum }}L_{\sigma }^{2}+\underset{\vec{i},\vec{j}}{\sum }J\left(\sigma_{\vec{i}},\;\sigma_{\vec{j}}\right)\left(1-\delta \left(\sigma_{\vec{i}},\;\sigma_{\vec{j}}\right)\right).$$ (1)

The first term models the compressibility of cells by penalising the deviation of cell areas from a target area *A*_0_. The second term represents the contractility of the cell cortex as a spring with zero equilibrium length (i.e., the target length of the cell perimeter is zero). The penalty parameter *λ*_cont_ represents cortical actomyosin contractility, around the lateral cell membrane (Reffay et al., 2014). The last term describes the cell-cell adhesion mediated by adhesion molecules, such as E-cadherin (Charras and Yap, 2018). *J* is the boundary energy cost at neighbouring lattice sites $\vec{i}$ and $\vec{j}$ . The Kronecker *δ* function prevents counting pixels that belong to the same cell. When both lattice sites $\vec{i}$ and $\vec{j}$ correspond to cells, $J\left(\sigma_{\vec{i}},\;\sigma_{\vec{j}}\right)=\lambda_{{\text{adh}}_{\text{cc}}}$ ; when one lattice site corresponds to cell and another site corresponds to the substrate $J\left(\sigma_{\vec{i}},\;\sigma_{\vec{j}}\right)=\lambda_{{\text{adh}}_{\text{cs}}}$ ; otherwise when one or both lattice sites represent empty space or boundary wall, the boundary energy cost *J* is set to zero. Note that $\lambda_{{\text{adh}}_{\text{cc}}}<0$ and $\lambda_{{\text{adh}}_{\text{cs}}}<0$ to represent that cells preferentially expand their boundaries shared with neighbouring cells or substrate. This is however balanced by the contractile tension along the cell cortex. The prefactors *λ*_area_, *λ*_cont_, and *λ*_adh_ reflect the relative importance of the corresponding cellular properties.

The dynamics of the CPM is defined by a stochastic series of elementary steps, where a cell expands or shrinks accommodated by a corresponding area change in the adjacent cell (or empty area) (Glazier and Graner, 1993; Swat et al., 2012). The algorithm randomly selects two adjacent lattice sites $\vec{i}$ and $\vec{j}$ , occupied by different cells $\sigma_{\vec{i}}\neq \sigma_{\vec{j}}$ . The elementary step is an attempt to copy $\sigma_{\vec{i}}$ into the adjacent lattice site $\vec{j}$ , which takes place with probability

$$P\left(\sigma_{\vec{i}}\to \sigma_{\vec{j}}\right)=\left\{\begin{array}{cc} 1\; & \text{for}\Delta E\leq 0\\ e^{-\Delta E/T}\; & \text{for}\Delta E>0 \end{array}\right.$$ (2)

where Δ*E* is the change in functional (1) due to the elementary step considered, and the temperature parameter *T* is an arbitrary scaling factor. A Monte Carlo step (MCS) of the simulation, the natural unit of time in the model, is set to *n* elementary steps–where *n* is the total number of lattice sites in the simulated area (Swat et al., 2012). Together, Eqs 1, 2 imply that cell configurations which increase the energy in functional (1) are less likely to occur. Thus, the cell population evolves through stochastic rearrangements in accordance with the biological dynamics incorporated into the effective energy function *E*.

Among multiple environmental factors that can regulate cell proliferation, cell growth factors and cell-substrate adhesion are most crucial (Schwartz and Assoian, 2001; Brakebusch et al., 2002): the probability of cell proliferation for individual cells increases with the cell area (Streichan et al., 2014) and substrate stiffness (Mohan et al., 2018).We therefore define cell proliferation probability as a function of cell area and adhesion to the substrate in the form of the Hill function, which is widely used in mathematical modelling of binding of molecular structures of cells (Santillán, 2008). At every MCS, if a cell *σ* reaches its target area (i.e., *A*_*σ*_ ≥ *A*_0_), the probability of proliferation is given by the following expression:

$$P_{\text{div}}=P_{\text{max}}\frac{n_{s}^{k}}{n_{s}^{k}+{\left(\gamma \sqrt{A_{0}}\right)}^{k}}$$ (3)

where *P*_max_ is the maximum probability of proliferation and *n*_*s*_ denotes the number of boundary pixels of a cell adjacent to the substrate, representing cell-substrate adhesion sites (Paddillaya et al., 2019). We assume that the Hill half-saturation threshold is given by the dimension in pixels of a square shaped cell, i.e., $\gamma \sqrt{A_{0}}$ with a multiplicative factor *γ*. Together, Eq. 3 expresses that the proliferation probability of a cell increases as the cell is more adhesive to the substrate. This is consistent with experiments (Chen et al., 1997; Provenzano and Keely, 2011; Mohan et al., 2018) where increased area of cell-substrate contact enhanced cell growth, and thus proliferation. Further, *P*_div_ = 0 for cells not adhered to the substrate, representing that cell proliferation can occur only on the substrate (Adam Hacking et al., 2013).

Our simulations are implemented using the open-source software package CompuCell3D (CC3D) (Swat et al., 2012). Each simulation starts with a single cell of the size 15 × 15 pixels placed on a substrate of width of 450 pixels and allowed to proliferate following Eq. 3. The simulation domain is surrounded by wall cells that prevent the cells from sticking to the lattice boundaries. The wall cells are excluded from participating in the pixel copies of the Potts model (Swat et al., 2009). If a cell division occurs, the cell is divided along a plane specified by a normal vector *n*_div_ = (*n*_*x*_, *n*_*z*_), where *n*_*x*_ and *n*_*z*_ are the components normal to the plane. The division then results in two cells each with area $\approx A_{0}/2$ . Then according to Eqs 1, 2 these two cells grow to reach the target area *A*_0_. Table 1 summarises the parameter values used in our computational simulations.

**TABLE 1:** Model parameters.

Parameter	Value
Initial cell size (pixel × pixel)	15 × 15
Initial cell area, *A*_*σ*_ (pixel × pixel)	225
Preferred area, *A*_0_ (pixel × pixel)	225
Temperature, *T*	50
Half-saturation constant, *γ*	2
Hill coefficient, *k*	10
Maximal proliferation probability, *P*_max_	0.1

## 3 Results and Discussion

### 3.1 Single Cell Morphology

Since the multicellular morphogenesis is partly driven by changes in the shape of individual cells (Widmann and Dahmann, 2009), our starting point for modelling collective epithelial cell morphology is to explore single-cell morphology in response to its mechanical properties, when cell proliferation is switched off. Typical snapshots of single-cell morphology in the steady-state are shown in Figure 1. We find that the squamous (i.e., flat)-to-cuboidal shape transition is promoted by increasing cell contractility. A similar shape transition is also found with decreasing cell-substrate adhesion; see Figures 1A,B.

To better understand how the single-cell morphology can influence the multi-cellular dynamics, we analyse the cell area and number of cell-substrate adhesion pixel (which affect the probability of cell proliferation) in response to the mechanical control parameters. This enables us to predict the combination of mechanical properties that can lead to different collective cell behaviors.

**FIGURE 2:**
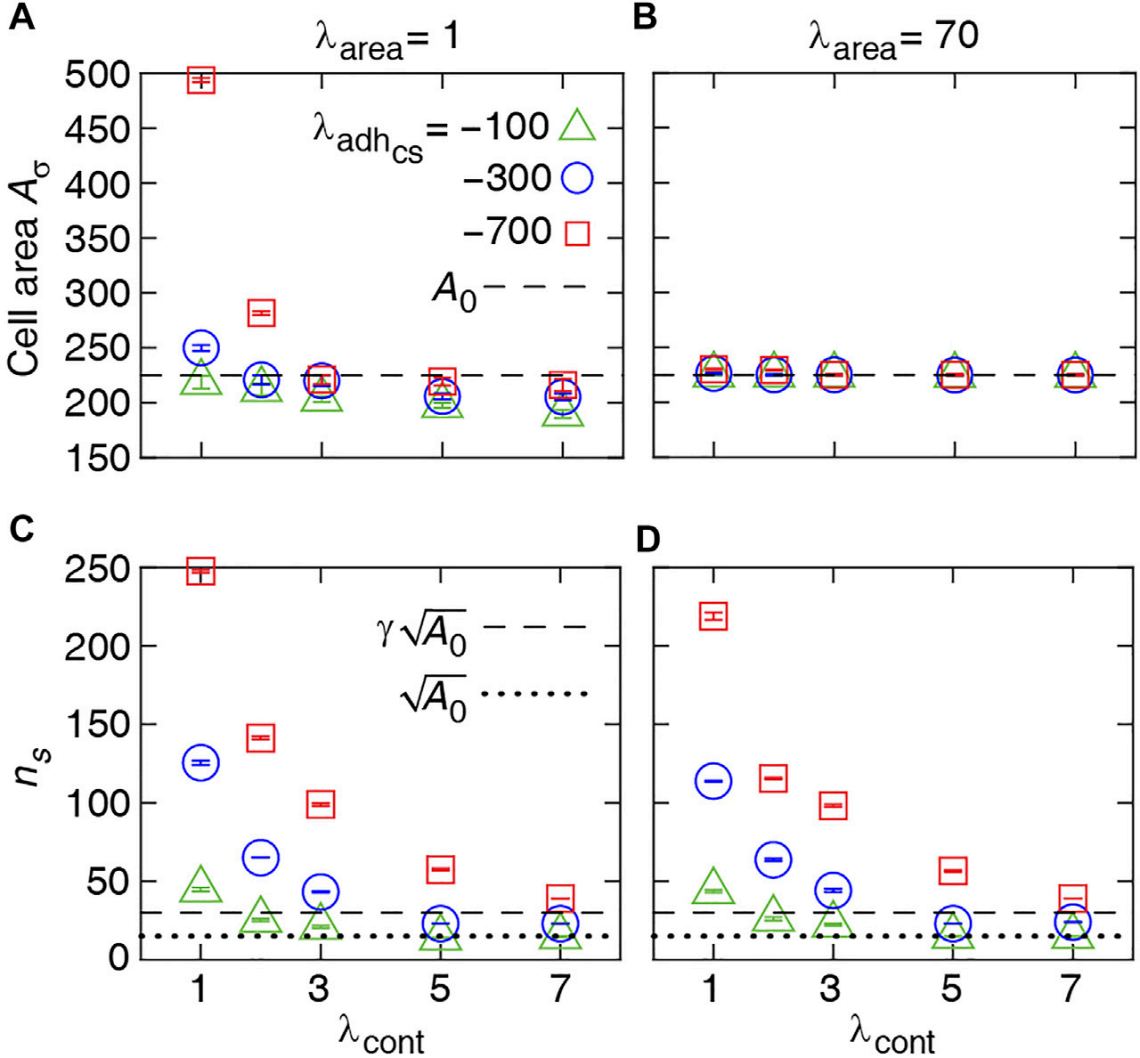
Numerical results for single-cell properties. **(A, B)** Area of the cell in steady state versus contractility strength *λ*_cont_ at various *λ*_area_ and cell-substrate adhesion $\lambda_{{\text{adh}}_{\text{cs}}}$ . **(C, D)** Number of cell-substrate adhesion sites *n*_*s*_ in the steady state versus *λ*_cont_ at various *λ*_area_ and $\lambda_{{\text{adh}}_{\text{cs}}}$ . Each symbol is derived from an individual simulation run and corresponds to mean ± SD.

Figure 2A shows that the average cell area increases with cell-substrate adhesion, which is more evident with weak cell contractility. Contrarily, with strengthening cell contractility and decreasing cell-substrate adhesion, the average area of a cell falls below its target area *A*_0_. On the other hand, with increasing *λ*_area_, the average cell area remains close to *A*_0_ at all *λ*_cont_ and $\lambda_{{\text{adh}}_{\text{cs}}}$ ; see Figure 2B. Further, increasing cell-substrate adhesion and weakening cell contractility expand cell-substrate adhesion sites; see Figures 2C,D. Together, these numerical results suggest that monolayers and multilayered structures are more likely to form with increasing cell-substrate adhesion and weaker cell contractility, due to increased proliferation probability of individual cells. Further, non-confluent structures are generated when cells have strong cortex contractility and low adhesion to the substrate.

**FIGURE 3:**
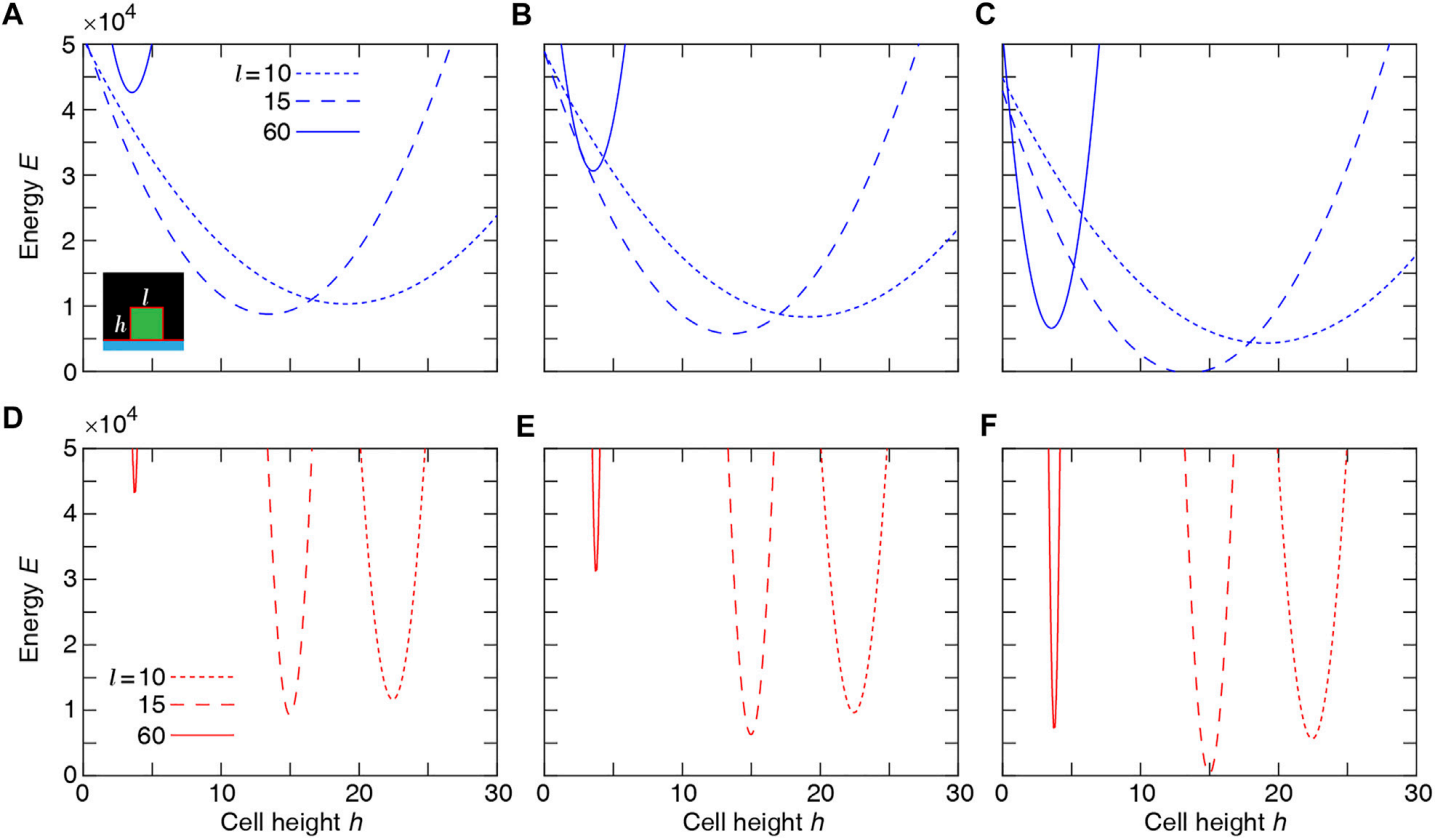
Single-cell Potts energy *E* versus cell height *h* at various cell length *l* calculated using Eq. 4. Top row: *λ*_area_ = 1 and *λ*_cont_ = 3 at $\lambda_{{\text{adh}}_{\text{cs}}}=-100$ **(A)**, −300 **(B)**, −700 **(C)**. Bottom row: *λ*_area_ = 70 and *λ*_cont_ = 3 at $\lambda_{{\text{adh}}_{\text{cs}}}=-100$ **(D)**, −300 **(E)**, −700 **(F)**.

**FIGURE 4:**
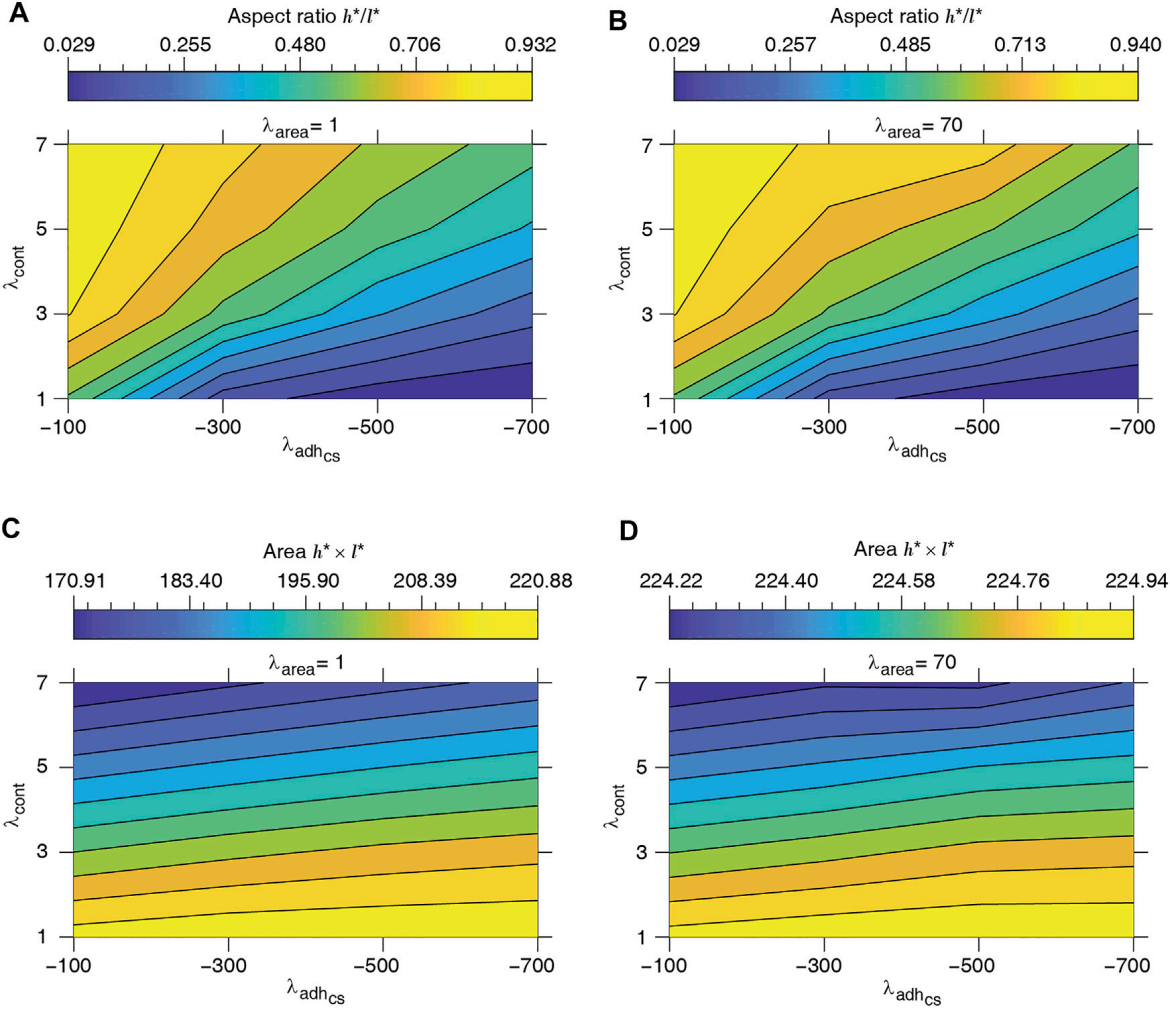
Equilibrium single-cell aspect ratio h*/l* **(A, B)** and area h* × l* **(C, D)** versus cell-substrate adhesion $\lambda_{{\text{adh}}_{\text{cs}}}$ and contractility *λ*_cont_. l* and h*: cell length and height at the mechanical equilibrium, respectively, corresponding to rectangular cell shape; see Eq. 4.

We check the consistency of these simulation results with the estimated energy minimum determined for a simplified rectangular cell shape. The energy function for a single rectangular cell reads:

$$E\left(l,h\right)={\text{λ}}_{\text{area}}{\left(lh-A_{\text{0}}\right)}^{2}+{\text{λ}}_{\text{cont}}{\left(2l+2h\right)}^{2}+{\text{λ}}_{{\text{adh}}_{\text{cs}}}l,$$ (4)

where *l* and *h* are cell length and height (see schematic inset, Figure 3). The minimum of the energy *E* is determined by solving the equations:

$$\frac{\partial E\left(l,h\right)}{\partial l}=0,\;\frac{\partial E\left(l,h\right)}{\partial h}=0,$$ (5)

which results in

$$\begin{array}{cc} 2{\text{λ}}_{\text{area}}h\left(lh-A_{0}\right)+8{\text{λ}}_{\text{cont}}\left(l+h\right)+{\text{λ}}_{{\text{adh}}_{\text{cs}}} & =0,\\ 2{\text{λ}}_{\text{area}}l\left(lh-A_{0}\right)+8{\text{λ}}_{\text{cont}}\left(l+w\right) & =0, \end{array}$$ (6)

to define cell length l* and height h* at mechanical equilibrium. The typical dependence of the energy function on the cell height and width is illustrated in Figure 3. Assuming that the mechanical equilibrium at steady state can be approximately estimated from the minimisation of the energy function corresponding to a rectangular cell (Eq. 4), we calculate the steady cell aspect ratio and area. The results shown in Figure 4 are consistent with the phase diagram of single-cell morphology in Figure 1 and also show qualitative agreement with the CPM simulation results in Figures 2A,B.

### 3.2 Collective Multicellular Morphology

We now use the model to simulate a system of proliferating cells various combinations of mechanical parameters. The simulations are started with a single cell placed on the substrate in the middle of the domain and cell division is allowed according to the rules described above, i.e., when the cell area is larger than *A*_0_ with a probability *P*_*div*_ dependent on the number cell adhesion sites (pixels) attached to the substrate.

During morphogenesis, oriented cell divisions are essential for the generation of cell diversity and for tissue shaping (Finegan and Bergstralh, 2019). The long-standing Hertwig’s rule (or the long axis rule) states that cells tend to divide at their cytoplasmic centre perpendicular to their longest axis (Hertwig, 1884). More recent studies have revealed that the proliferation is oriented by additional cellular properties, e.g., spatial distribution of the cell–substrate adhesion sites (Théry et al., 2005) and actomyosin-based mechanical tension dependent (LeGoff et al., 2013) and independent (Scarpa et al., 2018; Finegan and Bergstralh, 2019) of cell shape. However, current evidence on the role of cell shape and different sets of intracellular mechanisms in orienting cell proliferation remains inconclusive (Collinet and Lecuit, 2013; Finegan and Bergstralh, 2019). Our model allows a convenient way to simulate collective cell morphologies by considering different orientations of cell division axis and varying mechanical properties of cells in various combinations.

**FIGURE 5:**
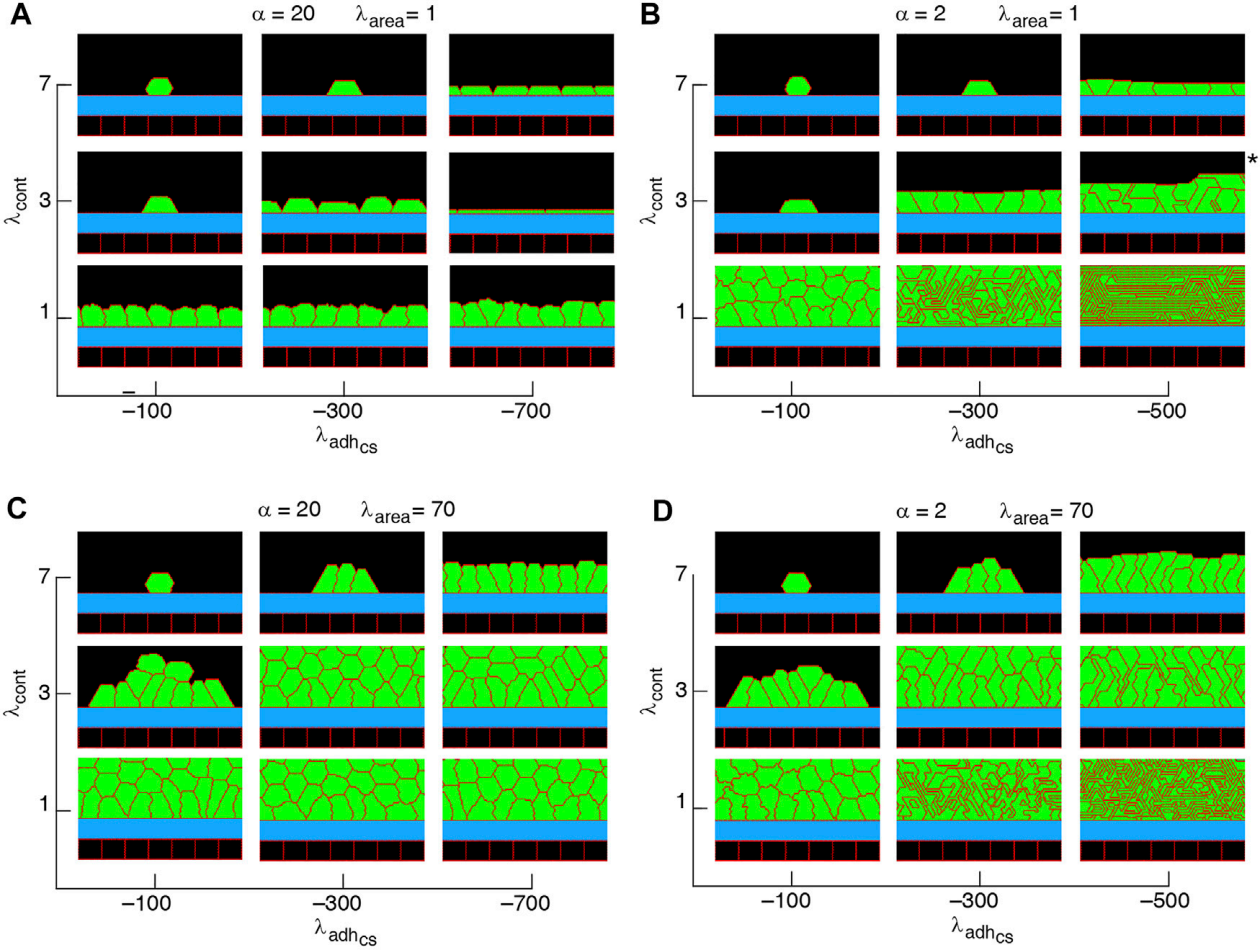
Phase diagram of steady state collective cell morphology when the axis of division is perpendicular to the substrate, i.e., *n*_div_ = (1, 0); see Eq. 3 **(A–D)**. $\alpha =\lambda_{{\text{adh}}_{\text{cs}}}/\lambda_{{\text{adh}}_{\text{cc}}}$ . *Slow-growing multilayers. See Supplementary Movies S1–S5.

**FIGURE 6:**
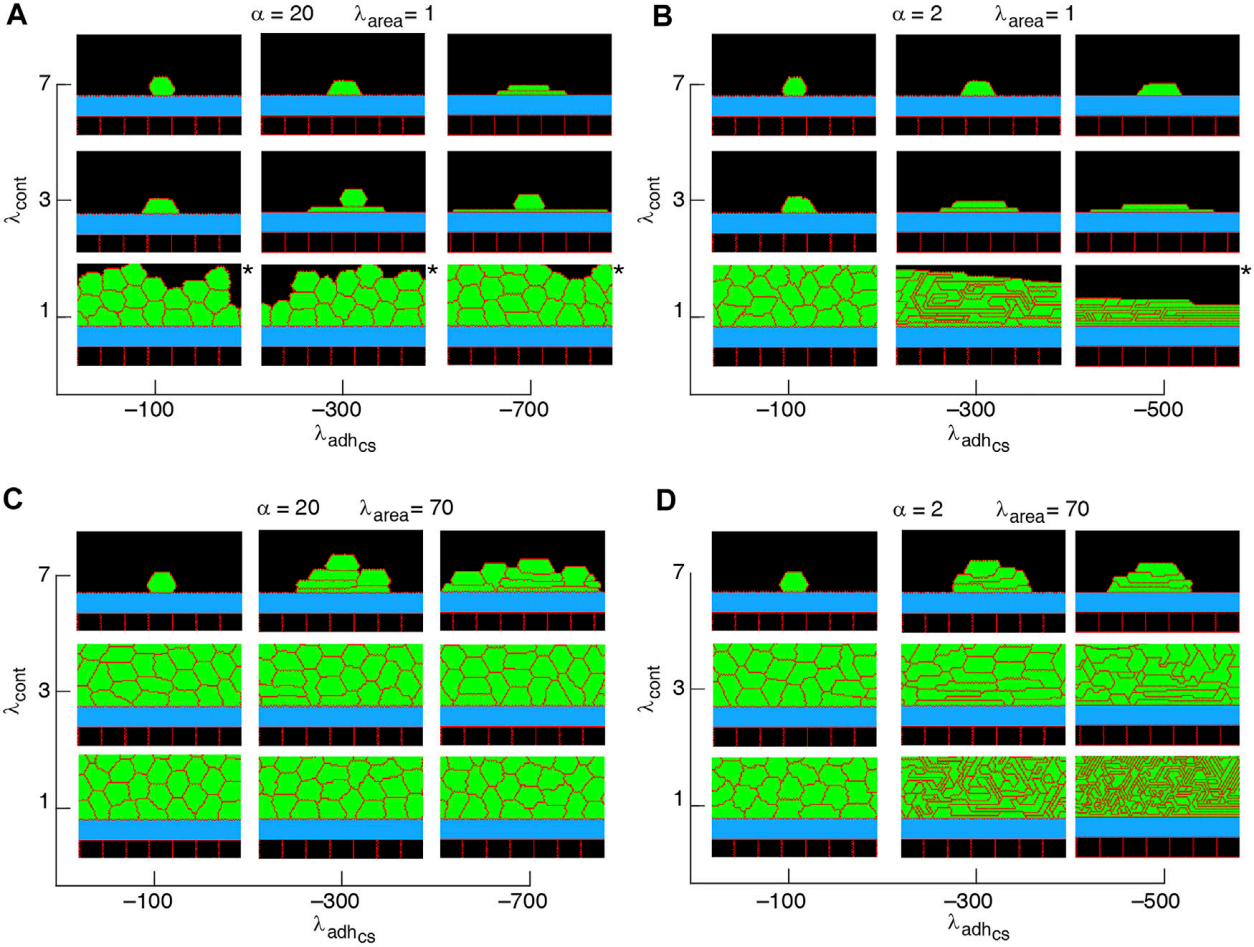
Phase diagram of collective cell morphology with horizontal orientation of cell proliferation, where the axis of division is parallel to the substrate, i.e., *n*_div_ = (0, 1); see Eq. 3 **(A–D)**. $\alpha =\lambda_{{\text{adh}}_{\text{cs}}}/\lambda_{{\text{adh}}_{\text{cc}}}$ . *Slow-growing multilayers. See Supplementary Movies S6, S7.

**FIGURE 7:**
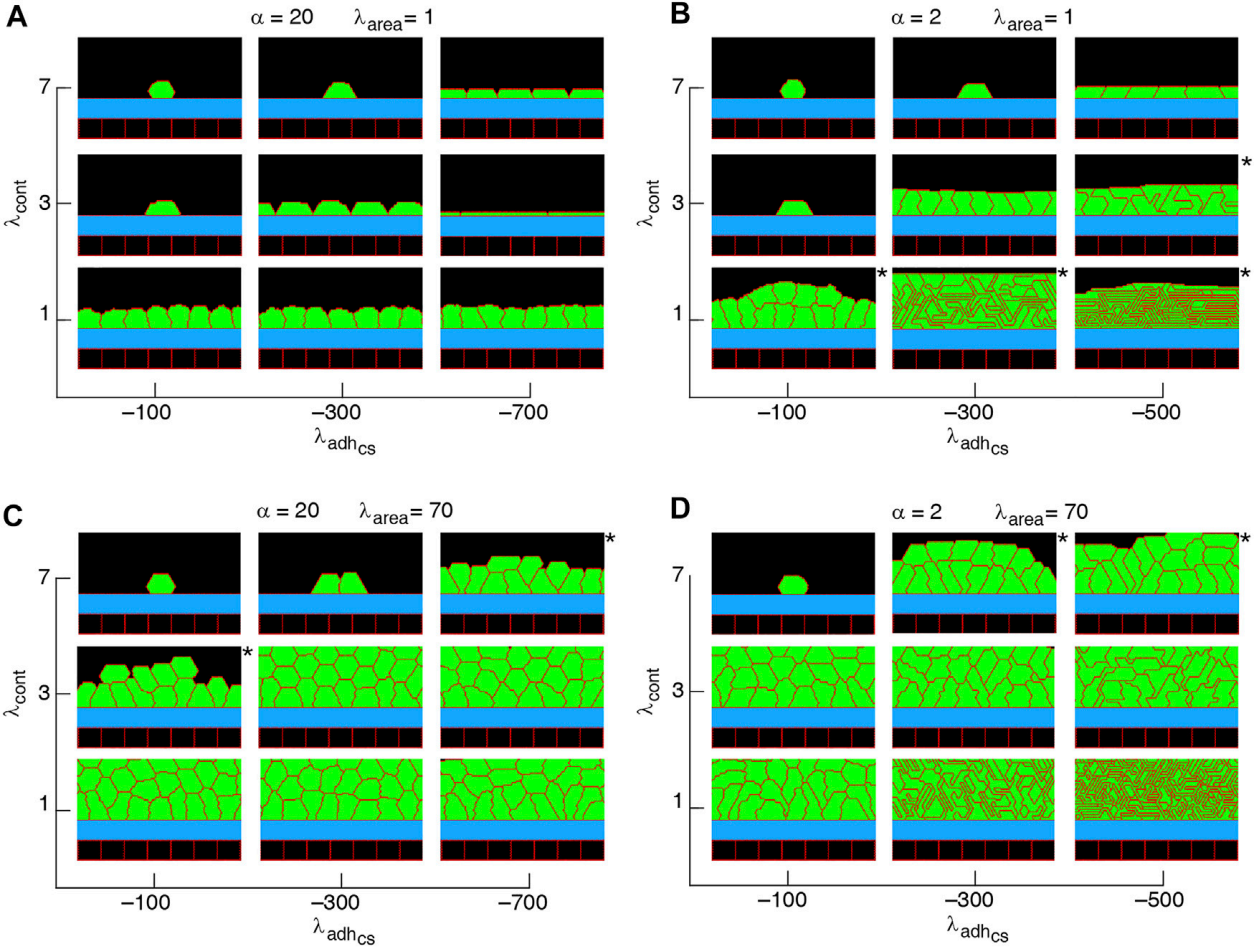
Phase diagram of collective cell morphologies when orientation of cell proliferation is along the major axis of the cells **(A–D)**. $\alpha =\lambda_{{\text{adh}}_{\text{cs}}}/\lambda_{{\text{adh}}_{\text{cc}}}$ . *Slow-growing multilayers. See Supplementary Movie S8.

Steady state phase diagrams of the collective morphology with horizontal, vertical, and random cell division orientation are presented in Figures 5–7(A–D), Supplementary Figure S1. First, we note that the cell shapes in the multicellular system in most cases can be quite different from the shape of a single isolated cell obtained for the same set of mechanical parameters. We observe three main types of multi-cellular structures and behaviors developing in the simulations: 1) For certain parameter combinations, the cell division is either completely blocked or is very limited resulting in the formation of a small group of cells without forming a confluent cell layer along the substrate over the whole domain. 2) A cell monolayer can form through repeated cell divisions in such a way that cell proliferation stops in a self-regulated manner once a fully confluent layer is formed. This layer may be composed of flat or tall cells. 3) In multi-layered structures, the cell division continues indefinitely (although it is still restricted to the basal cells along the substrate) and the height of the cell layer increases over time. In a real multilayered epithelium, the height of such layer can be controlled by differentiation and death of the non-proliferating cells that are not adhered to the substrate. Since we focus on the emergence of the different cell layer structures and the corresponding cell shapes, we do not include cell death and differentiation in our model. The simulation results also show that for high cell-substrate adhesion and low contractility ( $\lambda_{{\text{adh}}_{\text{cs}}}=-500$ and *λ*_cont_ = 1 in Figures 5B–7B,D) multicellular structures with irregular thin cell shapes develop. The formation of such structures is obviously not realistic and cannot appear in real tissues as it would be prevented by the internal cell cytoskeleton which is not included in the energy function of our model.

Non-confluent structures are formed at high cell contractility and reduced cell-substrate adhesion, independently of the proliferation orientation; see Figures 5–7, Supplementary Figure S1. This is due to reductions in both cell area and the number of cell-substrate adhesion sites in individual cells which reduce the probability of cell proliferation; see Figure 2.

**FIGURE 8:**
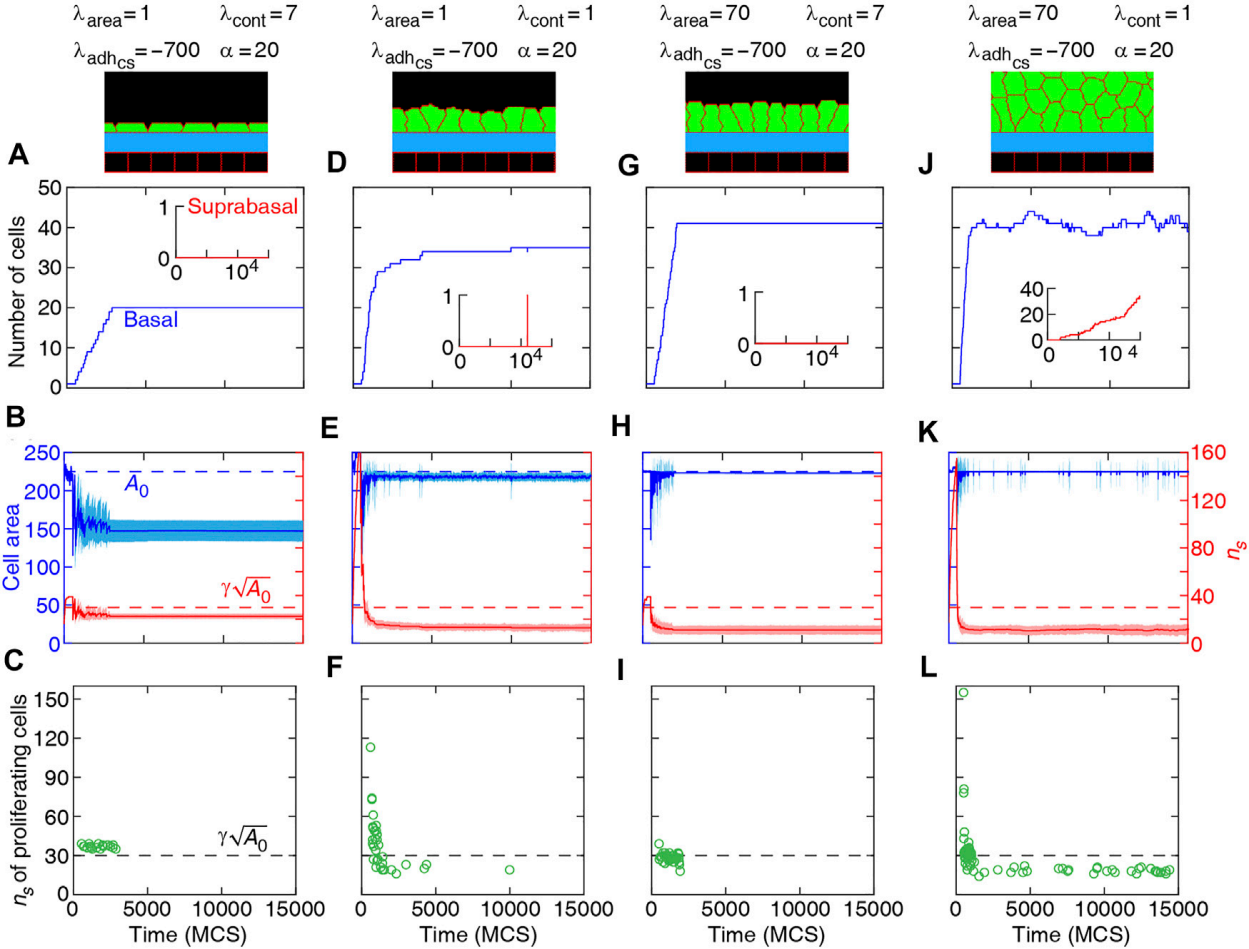
Dynamics of basal cells in four different collective morphologies illustrated with snapshots and associated mechanical parameters. Top row **(A,D,G,J)**: number of basal cells (with adherence to the substrate) and suprabasal cells (without adherence to the substrate) versus time. Middle row **(B,E,H,K)**: area (left axis) and number of cell-substrate adhesion sites *n*_*s*_ (right axis) for basal cells versus time. Solid curves: mean. Shaded region: SD. Bottom row **(C,F,I,L)**: *n*_*s*_ of proliferating cells versus time. Orientation of cell proliferation is vertical, *n*_div_ = (1, 0). $\alpha =\lambda_{{\text{adh}}_{\text{cs}}}/\lambda_{{\text{adh}}_{\text{cc}}}$ . See Supplementary Movie S1–S4.

Confluent monolayers and multilayered structures are formed with increasing cell-substrate adhesion and lowering cell contractility. With proliferation orientation perpendicular to the substrate, monolayers of squamous (flat), cuboidal, and columnar (tall) cells are found; see Figure 5. The expansion of monolayers typically happens through the division of border cells (at both edges of the monolayer) on the substrate, while the other cells inside the monolayer do not divide or only relatively rarely. Once the layer becomes confluent cell crowding limits the cell area and the cell-substrate adhesion sites, due to which the probability of proliferation decreases; see Figures 8A–I. At certain combinations of mechanical properties (summarised in Figure 5 and analysed in Figures 8A–I), cells stop proliferating once a confluent monolayer is formed see Supplementary Movies S1–S3, S5. Squamous cells are mostly found for high cell-substrate adhesion (relative to cell-cell adhesion), increased cortex contractility and reduced *λ*_area_ parameter. Increasing *λ*_area_, in this regime, leads to squamous-to-columnar shape transition.

A major factor that contributes to monolayer-to-multilayer transition is cell crowding. When the cell density cannot increase anymore in the basal layer, while cell deformations are likely, cells are extruded from the monolayer. Accordingly, cells at the basal layer can expand their area increasing the probability of their proliferation and further extrusion events; see Figures 8J–L and Supplementary Movie S4. Overall, monolayer-to-multilayer transition is more likely to appear with a combination of parameters that increase *λ*_area_, increasing cell-substrate adhesion, reduced cortical contractility, and with proliferation orientation being parallel to the substrate, random, or along the major axis of the cell; see Figures 6, 7, Supplementary Figure S1.

Our simulation results complement earlier findings on collective epithelial morphology. Simulation results characterise cell area strength *λ*_area_ as a major factor that influences collective morphology: increasing *λ*_area_ generates multilayer structures, whereas with reducing *λ*_area_ monolayer and non-confluent structures appear; see Figures 5–7, Supplementary Figure S1. Increasing *λ*_area_ promotes proliferation probability, by increasing cell area to approach the target area *A*_0_; see Eq. 1. At a low *λ*_area_, it is more likely that cell area deviates from *A*_0_ resulting in smaller probability of proliferation. This effect is evident in the generation of monolayers, where cell proliferation is limited by cell area and the number of cell-substrate adhesion sites; see Figures 8A–I. However, in multilayer structures, the growth of cell area is facilitated (by *λ*_area_, $\lambda_{{\text{adh}}_{\text{cs}}}$ , and *λ*_cont_) so that the crowding does not block cell proliferation events and continuous cell extrusions out of the basal layer lead to multilayered structure; see Figures 8J–L. At stronger cell-substrate adhesion $\lambda_{{\text{adh}}_{\text{cs}}}$ , the extruded cells may return back to the basal layer; see inset in Figure 8D. Further, cortex contractility *λ*_cont_ affects cell proliferation probability through influencing cell size and shape. With lowering *λ*_cont_ and strengthening cellular adhesion, cell shapes become softer (i.e., stretched with dynamic boundaries), in contrast to the more rounded cell shapes with approximately static cell boundaries at higher *λ*_cont_ (Farhadifar et al., 2007; Khataee et al., 2020; R. Noppe et al., 2015). These fluctuations in the cell size then increase the probability of proliferation in the model.

The simulation results are consistent with experimental observations. It has been observed that the probability of cell proliferation increases with cell area (Streichan et al., 2014) and reduction in cell area (imposed by mechanical constraints on tissue expansion) inhibits cell proliferation (Chen et al., 1997; Puliafito et al., 2012). Further, substrate stiffness has been known to be positively correlated with cell proliferation increasing substrate stiffness (dependent on cell-substrate adhesion) and was found to increase the proliferation rate (García et al., 1999; Provenzano and Keely, 2011; Mohan et al., 2018). It was shown that when the cell density cannot increase anymore in a monolayer (due to cell crowding), while the proliferation events still occur, newly generated cells are extruded out of the monolayer where they remained without adhering to the substrate (Deforet et al., 2014). These suprabasal cells may slide over the basal cells such that they migrate in and become basal cells themselves (Rognoni and Watt, 2018; Haensel et al., 2020). Experiments have also provided evidence that mechanical stretching stimulates cell proliferation (Aragona et al., 2013). The proliferation is activated in cells with flattened geometry where the cell growth is promoted, whereas in cells with round geometry, cell growth and thus proliferation are limited (Dupont et al., 2011; Aragona et al., 2013). It was suggested that the rounded cell geometry, compared to spread geometry, may differently affect the adhesion sites and their associated F-actin cytoskeleton (Low et al., 2014).

The simulated cell shapes in monolayers are also consistent with experimental observations. For example, with intermediate cortical contractility *λ*_cont_ = 3 and $\lambda_{{\text{adh}}_{\text{cs}}}=-300$ , increasing adhesion ratio *α* from 2 to 20 reduces cell height by factor 1.88; compare middle snapshots in Figures 5A,B. For squamous cells (*λ*_cont_ = 7 and $\lambda_{{\text{adh}}_{\text{cs}}}=-700$ ), cell height drops by factor 1.22; compare top right corner snapshots in Figures 5A,B. For columnar cells, a negligible reduction (by factor 1.10) in cell height is found with increasing *α*; compare top right corner snapshots in Figures 5C,D. These simulation results agree with experimental observations that lowering the lateral cell–cell adhesion decreases cell height (Weber et al., 2007; Melani et al., 2008; Montell, 2008; Gomez et al., 2012). It is also consistent with the theoretical prediction that the cell-cell lateral adhesion is a crucial parameter to increase cell height (Hannezo et al., 2014; Dasbiswas et al., 2018).

Our results show that with altering the proliferation orientation from being perpendicular to the substrate to be along the major axis of the cells, monolayers of columnar cells transition into multilayered structures; compare Figures 5, 7C,D, top right corner. With proliferation orientation perpendicular to the substrate, the new daughter cells are positioned to either left or right of the mother cell on the substrate. This way, cell crowding decreases the proliferation probability (see Eq. 3) and a monolayer of columnar cells are formed. However, when the proliferation orientation is along the major axis of the cells, new daughter cells can extrude from the basal layer, even before a confluent monolayer is formed (see Supplementary Movie S8). These extrusions then do not contribute to cell crowding on the basal layer and allow the basal cells to grow, so that further proliferation events occur and multilayered structures are formed. This is consistent with experimental observations (Chanet et al., 2017) showing that cell rounding is required for the division of columnar epithelial cells and without the cell rounding, cells remain elongated due to tight cell packing.

## 4 Conclusion

In this article, we introduced a 2D computational model to analyse the emergence of collective morphology of epithelial cells. The model allowed us to simulate diverse collective morphology using various combinations of mechanical properties of cells and the orientation of cell division axis. Our results suggest that non-confluent structures transition into confluent monolayers and multilayers with weakening cell contractility (*λ*_cont_) and strengthening cell-substrate adhesion $\left(\lambda_{{\text{adh}}_{\text{cs}}}\right)$ , due to increase in probability of cell proliferation. Confluent monolayers of squamous, cuboidal, and columnar cells are formed with proliferation axis perpendicular to the substrate. It is further suggested that monolayer-to-multilayer transition occurs by cell extrusion from the basal layer as a result of the interplay between mechanical parameters (*λ*_area_, *λ*_cont_, and $\lambda_{{\text{adh}}_{\text{cs}}}$ ) and the orientation of cell proliferation. Taken together, our simulation results suggest that desirable biomechanical features of individual cells can regulate multicellular tissue morphology.

The extension of the energy function of the 2D model to 3D is relatively straightforward [e.g., see (Hannezo et al., 2014)], but including the third dimension would significantly increase the computational cost of the simulations. We expect that for most cases the computational results on the multicellular morphology would be at least qualitatively similar since the cell shapes are isotropic within the plane of the substrate (i.e., *x*-*y* plane). In addition, the cell proliferation steps defined here take into account the progressive increase of cell volume (through growing of lateral area of cell *A*_*σ*_ and approaching *A*_0_) and the apical perimeter of the cells (through $\sqrt{A_{0}}=l$ in Eq. 3). Further, the presented 2D model is more amenable to efficient simulations (i.e., when performing parameter sweeps) and mathematical analysis, compared with that in 3D.

## Data Availability

The theoretical model in this study is included within the article.
