# Supplementary material for: Modelling the Collective Mechanical Regulation of the Structure and Morphology of Epithelial Cell Layers
Source: Front Cell Dev Biol. 2022 Mar 24;10:767688. doi: 10.3389/fcell.2022.767688 (PMC8987200; doi:10.3389/fcell.2022.767688)
Supplement: Supplementary file 4 [file DataSheet1.pdf]

Supplementary Material

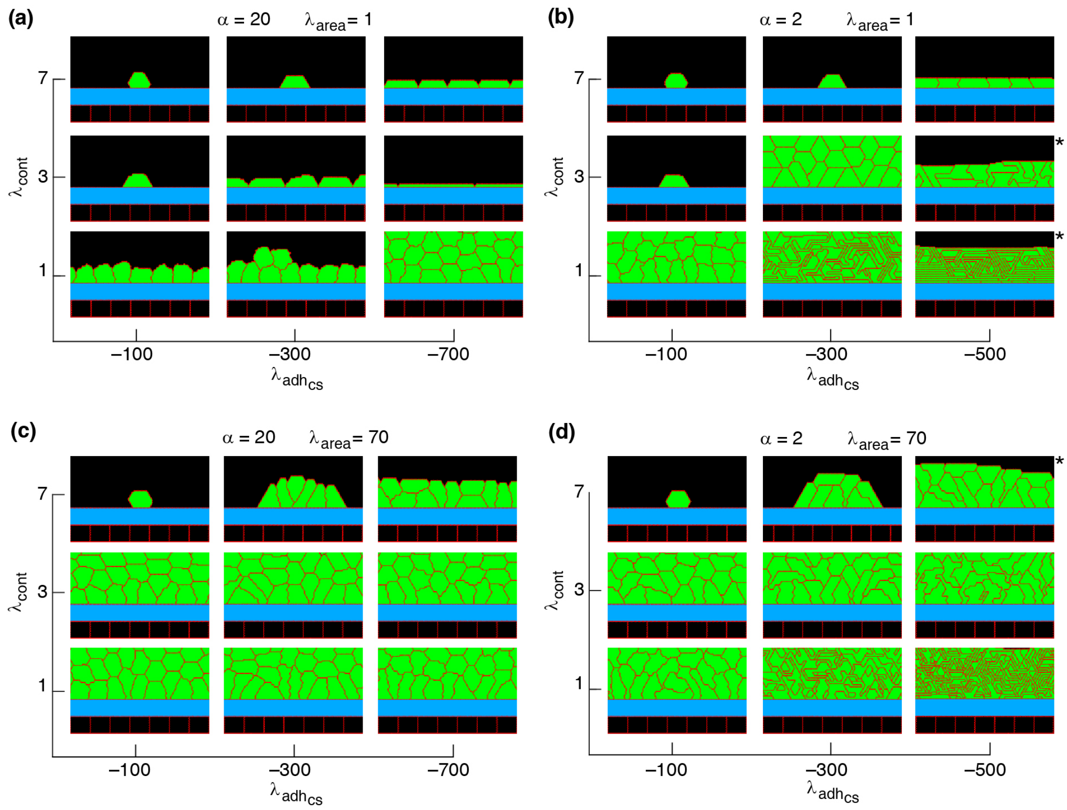

Figure S1: Phase diagram of collective cell morphologies with random orientation of cell proliferation. $\alpha = \lambda_{\mathrm{adh_{cs}}}/\lambda_{\mathrm{adh_{cc}}}$. *Slow-growing multilayers. See Movies 9, 10.

Movie captions

Movie 1. A simulation of a monolayer of squamous cells, where the axis of cell proliferation is perpendicular to the substrate, i.e., $n_{\text{div}} = (1, 0)$; see Equation (3). Simulation parameters are: $\lambda_{\text{area}} = 1$, $\lambda_{\text{cont}} = 7$, $\lambda_{\text{adh}_{\text{cs}}} = -700$, and $\lambda_{\text{adh}_{\text{cc}}} = -35$.

Movie 2. A simulation of a monolayer of cuboidal cells, where the axis of cell proliferation is perpendicular to the substrate, i.e., $n_{\text{div}} = (1, 0)$; see Equation (3). Simulation parameters are: $\lambda_{\text{area}} = 1$, $\lambda_{\text{cont}} = 1$, $\lambda_{\text{adh}_{\text{cs}}} = -700$, and $\lambda_{\text{adh}_{\text{cc}}} = -35$.

Movie 3. A simulation of a monolayer of columnar cells. Simulation parameters are: $\lambda_{\text{area}} = 70$, $\lambda_{\text{cont}} = 7$, $\lambda_{\text{adh}_{\text{cs}}} = -700$, and $\lambda_{\text{adh}_{\text{cc}}} = -35$. The axis of cell proliferation is perpendicular to the substrate, i.e., $n_{\text{div}} = (1, 0)$; see Equation (3).

Movie 4. A simulation of a multilayer structure, where the axis of cell proliferation is perpendicular to the substrate, i.e., $n_{\text{div}} = (1, 0)$; see Equation (3). Simulation parameters are: $\lambda_{\text{area}} = 70$, $\lambda_{\text{cont}} = 1$, $\lambda_{\text{adh}_{\text{cs}}} = -700$, and $\lambda_{\text{adh}_{\text{cc}}} = -35$.

Movie 5. A simulation of a monolayer of cuboidal cells, where the axis of cell proliferation is perpendicular to the substrate, i.e., $n_{\text{div}} = (1, 0)$; see Equation (3). Simulation parameters are: $\lambda_{\text{area}} = 1$, $\lambda_{\text{cont}} = 1$, $\lambda_{\text{adh}_{\text{cs}}} = -100$, and $\lambda_{\text{adh}_{\text{cc}}} = -5$.

Movie 6. A simulation of a non-confluent structure. Simulation parameters are: $\lambda_{\text{area}} = 1$, $\lambda_{\text{cont}} = 3$, $\lambda_{\text{adh}_{\text{cs}}} = -300$, and $\lambda_{\text{adh}_{\text{cc}}} = -15$. The axis of cell proliferation is parallel to the substrate, i.e., $n_{\text{div}} = (0, 1)$; see Equation (3).

Movie 7. A simulation of a multilayer structure, where the axis of cell proliferation is parallel to the substrate, i.e., $n_{\text{div}} = (0, 1)$; see Equation (3). Simulation parameters are: $\lambda_{\text{area}} = 70$, $\lambda_{\text{cont}} = 3$, $\lambda_{\text{adh}_{\text{cs}}} = -300$, and $\lambda_{\text{adh}_{\text{cc}}} = -150$.

Movie 8. A simulation of a monolayer, where the axis of proliferation is along the major axis of the cells. Simulation parameters are: $\lambda_{\text{area}} = 70$, $\lambda_{\text{cont}} = 7$, $\lambda_{\text{adh}_{\text{cs}}} = -700$, and $\lambda_{\text{adh}_{\text{cc}}} = -35$.

Movie 9. A simulation of a monolayer of squamous cells, with random orientation of cell proliferation. Simulation parameters are: $\lambda_{\text{area}} = 1$, $\lambda_{\text{cont}} = 3$, $\lambda_{\text{adh}_{\text{cs}}} = -300$, and $\lambda_{\text{adh}_{\text{cc}}} = -15$.

Movie 10. A simulation of a multilayer structure, with random orientation of cell proliferation. Simulation parameters are: $\lambda_{\text{area}} = 70$, $\lambda_{\text{cont}} = 3$, $\lambda_{\text{adh}_{\text{cs}}} = -300$, and $\lambda_{\text{adh}_{\text{cc}}} = -150$.
